# Resting Heart Rate and Long-Term Outcomes in Patients with Percutaneous Coronary Intervention: Results from a 10-Year Follow-Up of the CORFCHD-PCI Study

**DOI:** 10.1155/2019/5432076

**Published:** 2019-04-01

**Authors:** Ying-Ying Zheng, Ting-Ting Wu, You Chen, Xian-Geng Hou, Yi Yang, Xiang Ma, Yi-Tong Ma, Jin-Ying Zhang, Xiang Xie

**Affiliations:** ^1^Department of Cardiology, First Affiliated Hospital of Zhengzhou University, Key Laboratory of Cardiac Injury and Repair of Henan Province, Zhengzhou 450052, China; ^2^Department of Cardiology, First Affiliated Hospital of Xinjiang Medical University, Urumqi 830011, China

## Abstract

**Background:**

The relationship between heart rate in CAD patients who underwent percutaneous coronary intervention (PCI) and had long-term outcomes over up to 10 years of follow-up has not been investigated.

**Methods:**

All patients were from the CORFCHD-PCI, a retrospective cohort study that included a total of 6050 CAD patients who underwent PCI from January 2008 to December 2016. One patient was excluded due to a lack of heart rate data. Ultimately, 6049 patients were enrolled. The primary outcome was long-term mortality after PCI.

**Results:**

Patients were divided into 5 groups according to heart rate quintiles: 1st quintile (heart rate <66 beats/min; *n*=1123), 2nd quintile (heart rate ≥66 beats/min to 72 beats/min; *n*=1010), 3rd quintile (heart rate ≥72 beats/min to 78 beats/min; *n*=1442), 4th quintile (heart rate ≥78 beats/min to 84 beats/min; *n*=1211), and 5th quintile (heart rate ≥84 beats/min; *n*=1263). After multivariate Cox regression analyses, the respective risks of ACM, CM, and MACEs were increased 79.1% (hazard risk (HR) = 1.791, 95% CI: 1.207–2.657, *P*=0.004), 56.9% (HR = 1.569, 95% CI: 1.019–2.416, *P*=0.041), and 25.5% (HR = 1.255, 95% CI: 0.990–1.590, *P*=0.060) in the 4th quintile and 98.7% (HR = 1.987, 95% CI: 1.344–2.937, *P*=0.001), 98.8% (HR = 1.988, 95% CI: 1.310–3.016, *P* < 0.001), and 0.36.1% (HR = 1.361, 95% CI: 1.071–1.730, *P*=0.012) in the 5th quintile compared with those in the 1st quintile. Patients with a heart rate of ≥80 beats/min had 89.4%, 115.2%, and 39.1% increased risk of ACM, CM, and MACEs, respectively, compared to those patients with a heart rate of <80 beats/min.

**Conclusion:**

The present study indicated that the resting heart rate is an independent predictor of adverse long-term outcomes in CAD patients who underwent PCI.

## 1. Introduction

Previous studies demonstrated that resting heart rate is a risk factor for mortality and adverse events in coronary artery disease with left ventricular dysfunction and ST-segment elevation acute myocardial infarction (STEMI) [1–4]. Recently, O'Brien and colleagues observed an association of the heart rate with outcomes after percutaneous coronary intervention (PCI) and found that the heart rate immediately before PCI is an independent predictor of adverse 30-day cardiovascular outcomes [5]. Most studies that evaluated the association of the heart rate with outcomes in STEMI patients have included a follow-up duration of up to 6 months [5–9]. The relationship between heart rate and long-term outcomes of patients with PCI has not been thoroughly investigated. Antoni et al. reported a relationship between heart rate and 1-year and 4-year outcomes in STEMI patients after PCI. The authors suggested that, in STEMI patients treated with primary PCI, heart rate at discharge was an important predictor of mortality during a follow-up for up to 4 years, even after adjustment for parameters reflecting a greater infarct size and the presence of heart failure [10]. However, Antoni et al. [10] included only 1453 patients, and the longest follow-up duration was 4 years. No studies with a larger sample size or longer duration of follow-up have been performed in recent years. Accordingly, in the present study, we aimed at investigating the relationship between heart rate before PCI and clinical outcomes for up to 10 years in patients with CAD who underwent PCI.

## 2. Methods

### 2.1. Study Design and Population

All patients were from the Clinical Outcomes and Risk Factors of Patients with Coronary Heart Disease after PCI (CORFCHD-PCI) study, which is a large, single-center retrospective cohort study based on case records and follow-up registry performed at the First Affiliated Hospital of Xinjiang Medical University. The details of the design have been registered on http://www.chictr.org.cn (Identifier: ChiCTR-ORC-16010153). In brief, the CORFCHD-PCI study was designed to evaluate the clinical outcomes and risk factors of CAD patients after PCI. We collected demographic data, clinical characteristics, risk factors, blood samples, biochemical data, ECG data, echocardiography, coronary angiography and PCI procedures, and short-term and long-term outcomes for CAD patients who underwent PCI at the First Affiliated Hospital of Xinjiang Medical University between January 2008 and December 2016.

**Figure 1:**
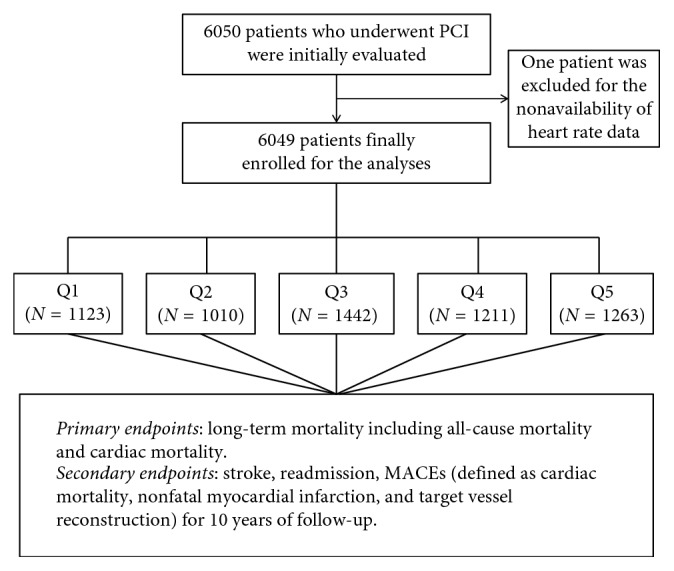
The flowchart of participant's inclusion.

In the present study, a total of 6050 patients with CAD after PCI were evaluated initially. One patient was excluded due to a lack of heart rate data. Ultimately, 6049 patients were enrolled in the present study.  Figure 1 shows the flowchart of inclusion and exclusion of the participants. The study protocol was approved by the ethics committee of the First Affiliated Hospital of Xinjiang Medical University. Because of the retrospective design of the study, the need to obtain informed consent from eligible patients was waived by the ethics committee.

### 2.2. Endpoints

The primary endpoints of the present study were long-term all-cause mortality (ACM) and cardiac mortality (CM). The secondary endpoints included stroke, bleeding events, readmission, and the major adverse cardiac events (MACEs), defined as the combination of cardiac death, recurrent myocardial infarction, and target vessel reconstruction.

### 2.3. Data Collection

Demographic data, cardiovascular risk factors, coronary angiography and PCI procedure indices, and laboratory data for all patients were recorded. The aim of the present study was to assess the relationship between heart rate and outcomes over up to 10 years of follow-up. For this purpose, resting heart rate was measured from 12-lead electrocardiography before PCI. We recorded cardiovascular risk factors, such as smoking, drinking, and history of diabetes mellitus and hypertension. We also collected the medical history and medication information, including antiplatelet therapy, CCBs, ACEIs or ARBs, *β*-blockers, and statins. Fasting blood samples were collected before coronary angiography. Serum concentrations of electrolytes, liver function, renal function, the myocardial enzyme profile, and blood glucose were measured using standard methods in the Central Laboratory of the First Affiliated Hospital of Xinjiang Medical University.

### 2.4. Statistical Analyses

Continuous data are presented as the mean ± standard deviation (mean ± SD). Categorical data are presented as frequencies and percentages. Heart rate was analyzed as a continuous variable categorized into five groups by quintiles (<66, 66–72, 72–78, 78–84, and ≥84 beats/min). All analyses were performed using SPSS 22.0 for Windows statistical software (SPSS Inc., Chicago, IL, USA). The differences between normally distributed numeric variables were analyzed by one-way ANOVA, while nonnormally distributed variables were analyzed by the Mann–Whitney *U* test or Kruskal–Wallis variance analysis as appropriate. The chi-square (*χ*^2^) test was employed for the comparison of categorical variables. Kaplan–Meier analysis was used for cumulative incidence rates of long-term outcomes, and the log-rank test was used for comparisons between groups. Multivariable analysis was performed to assess the predictive value of heart rate for outcomes over up to 10 years of follow-up. Hazard ratios (HRs) and 95% confidence intervals (CIs) were calculated. *P* < 0.05 was considered indicative of significance.

## 3. Results

### 3.1. Baseline Data

A total of 6049 patients, including 2044 ACS patients and 4005 stable CAD patients, were divided into 5 groups according to heart rate quintiles: 1st quintile (heart rate <66 beats/min; *n*=1123), 2nd quintile (heart rate ≥66 beats/min to 72 beats/min; *n*=1010), 3rd quintile (heart rate ≥72 beats/min to 78 beats/min; *n*=1442), 4th quintile (heart rate ≥78 beats/min to 84 beats/min; *n*=1211), and 5th quintile (heart rate ≥84 beats/min, *n*=1263). Baseline data are shown in Table 1. In the total population, a number of variables were significantly different between these five groups, including age, SBP, DBP, hypertension, diabetes, and use of *β*-blockers, aspirin, and statins (all *P* < 0.05). We did not find significant differences among these groups in therapy with calcium channel blockers (CCBs), ACEIs or ARBs, clopidogrel, LVEDD, LVEF, smoking, alcohol drinking, BMI, BUN, Cr, GLU, UA, TC, TG, HDL-C, LDL-C, ApoA1, ApoB, or Lp(a) (all *P* > 0.05). In addition, several characteristics of lesions and some PCI parameters between these 5 groups showed significant differences, such as CTO and multivessel lesions. In the ACS patients, we also found significant differences among these 5 groups in diabetes, hypertension, age, SBP, DBP, HR, CTO, and multivessel lesions. In the stable CAD patients, significant differences were observed among these 5 groups in diabetes, hypertension, age, SBP, DBP, HR, CTO, multivessel lesions, and treatment with clopidogrel and aspirin.

### 3.2. Clinical Outcomes

As shown in Table 2, there were 309 cases of all-cause mortality (ACM) during the follow-up. In total, the incidence of ACM in the 1st quintile was 43 (3.8%), the 2nd quintile was 38 (3.8%), the 3rd quintile was 62 (4.3%), the 4th quintile was 77 (6.4%), and the 5th quintile was 89 (7.0%). The ACM incidence was significantly higher in the 4th and 5th quintiles compared to that in the 1st quintile (both *P* < 0.001). We also found that CM occurred in 251 patients: 37 (3.3%) in the 1st quintile, 26 (2.6%) in the 2nd quintile, 48 (3.3%) in the 3rd quintile, 60 (5.0%) in the 4th quintile, and 80 (6.3%) in the 5th quintile. There was a significant difference in the CM incidence among these 5 groups (*P* < 0.001). Regarding the secondary endpoints, we found a significant difference among these 5 groups only in the incidence of MACEs (*P*=0.018). There were no significant differences among the groups in the incidence of stroke, readmission, or bleeding events (all *P* > 0.05). In the ACS patients, we found that there were significant differences among these groups in the incidence of ACM, CM, MACEs, and stroke. However, in the stable CAD patients, we found that there were significant differences among these groups only in the incidence of ACM and CM.

**Figure 2:**
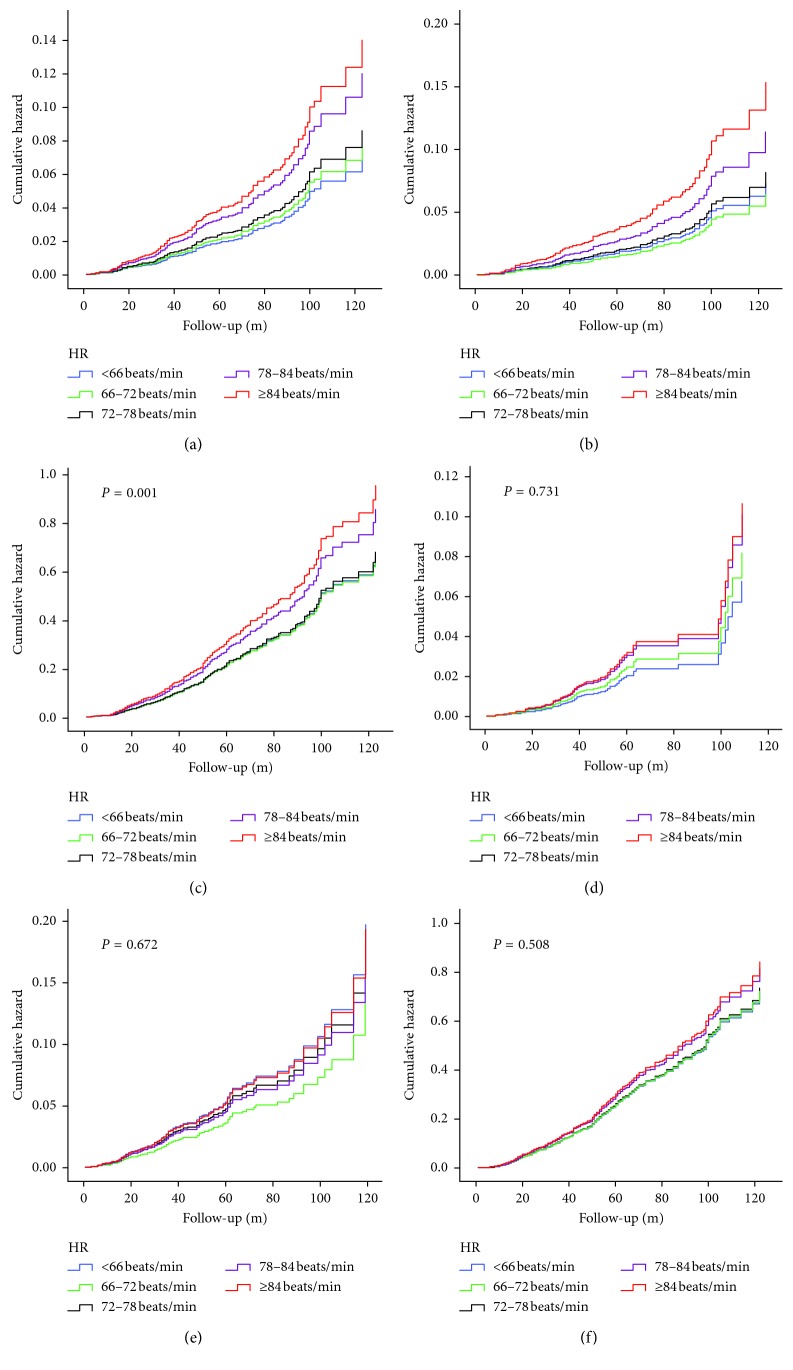
Cumulative Kaplan–Meier estimates of the time to the first adjudicated occurrence of primary endpoint and secondary endpoints: (a) ACM; (b) CM; (c) MACEs; (d) stroke; (e) bleeding; (f) readmission.

Kaplan–Meier curves for heart rate divided by quintiles and adverse outcomes are shown in Figure 2. Patients in the 4th quintile with a heart rate of 78–84 beats/min and the 5th quintile with a heart rate of 84 beats/min or higher showed significantly higher event rates for ACM (6.4% vs. 3.8% and 7.0% vs. 3.8%), CM (5.0% vs. 3.3% and 6.3% vs. 3.3%), and MACEs (14.9% vs. 12.2% and 14.5% vs. 12.2%) compared with patients in the lowest quintile with a heart rate lower than 66 beats/min. These differences were also found in both ACS patients and stable CAD patients (data not shown).

Multivariable analysis was performed to assess the prognostic value of heart rate for adverse outcome after adjusting for age, the presence of hypertension, diabetes, SBP, DBP, CTO, multivessel lesions, LVEDD, and LVEF, as well as therapy with *β*-blockers, aspirin, and statins. After multivariate Cox regression analyses, the respective risks of ACM, CM, and MACEs were increased 79.1% (hazard risk (HR) = 1.791, 95% CI: 1.207–2.657, *P*=0.004), 56.9% (HR = 1.569, 95% CI: 1.019–2.416, *P*=0.041), and 25.5% (HR = 1.255, 95% CI: 0.990–1.590, *P*=0.060) in the 4th quintile and 98.7% (HR = 1.987, 95% CI: 1.344–2.937, *P*=0.001), 98.8% (HR = 1.988, 95% CI: 1.310–3.016, *P* < 0.001), and 0.36.1% (HR = 1.361, 95% CI: 1.071–1.730, *P*=0.012) in the 5th quintile compared to those in the 1st quintile (Tables 3–5).

As shown in Table 6, analyses with heart rate as a continuous variable showed that every increase of 1 beat/min resulted in a significantly increased risk of 2.7%, 3.0%, and 1.7% for ACM, CM, and MACEs, respectively. In addition, compared to a heart rate of less than 80 beats/min, a heart rate ≥80 beats/min conferred 89.4%, 115.2%, and 39.1% increased risk of ACM, CM, and MACEs, respectively. These trends were also observed in both the ACS patients and stable CAD patients.

## 4. Discussion

In the present study, we demonstrate that resting heart rate in CAD patients treated with PCI was an independent predictor of adverse outcomes over up to 10 years of follow-up. The present results indicate for the first time the strong relationship between heart rate and adverse outcomes over a follow-up period of up to 10 years in CAD patients who underwent PCI.

Previously, many studies that investigated the relationship between heart rate and prognosis of CAD focused on CAD patients with left ventricular dysfunction or with the onset of acute coronary syndrome (ACS) [1–4, 11]. Few studies have focused on the relationship between heart rate and outcomes in stable CAD with PCI. Recent research performed by O'Brien et al. [5] reported that heart rate immediately before PCI is an independent predictor for the 30-day outcome. However, the follow-up period in the study by O'Brien et al. lasted only 30 days. The association between heart rate and long-term outcomes of patients remains unclear. A study by Bordejevic et al. [9] suggested that heart rate ≥80 beats/min in STEMI patients at admission is associated with a higher risk of in-hospital death, even after primary PCI. Wang et al. [12] investigated the effect of heart rate on the prognosis of ACS patients in a Chinese population and found that an elevated resting heart rate ≥61 beats/min was associated with an increased risk of MACEs over a one-year period. Jensen et al. [4] enrolled 2029 SCAD or ACS patients who underwent PCI to observe the relationship between heart rate and outcomes after 7 days and 2 years of follow-up. The authors found that an elevated discharge heart rate was independently associated with poor prognosis. However, the longest duration of follow-up was 4 years, and the sample size was relatively small in all of these published studies. In our study, we enrolled 6049 CAD patients who underwent PCI, and the time of follow-up was up to 10 years. To the best of our knowledge, this is the first study to demonstrate the relationship between heart rate and adverse outcomes over up to 10 years of follow-up in CAD patients who underwent PCI.

In clinical practice, beta-blockers are recommended for the secondary prevention of CAD [13–15]. Heart rate influences myocardial oxygen demand and supply, and a higher heart rate will increase myocardial oxygen consumption [16, 17]. Therefore, therapy for heart rate reduction may benefit patients with CAD. Recently, the association between heart rate and outcome has been investigated extensively. Noman et al. [18] performed a retrospective analysis of prospectively collected data on 2310 PPCI-treated STEMI patients and found that an elevated admission heart rate was associated with long-term all-cause mortality. Furthermore, beta-blocker therapy improved postdischarge survival in patients with an elevated admission heart rate. In Noman et al.'s study, the proportion of patients on beta-blocker therapy was 84.0%. However, in our study, only 40.3% of patients were administered beta-blockers. Although beta-blockers are recommended for the secondary prevention of CAD, the proportion of beta-blocker users was very low in our study. The relatively low compliance may be a possible explanation for the relatively low use of beta-blockers in the study cohort. In addition, we also found that there was a significant difference among these 5 groups in beta-blocker usage. However, after adjustment for beta-blockers, the heart rate remains an independent predictor of adverse outcomes, which indicated that the results in our study were not influenced by the usage of beta-blockers.

In our study, comparing heart rate quintiles, we found that only patients in the two highest quintiles (78–84 beats/min, ≥84 beats/min) were at increased risk of adverse outcomes. After adjustments for age, hypertension, diabetes, SBP, DBP, and therapy with beta-blockers, CCBs, and statins, the differences remained significant. We did not find a significant difference between the heart rate <66 beats/min, 66–72 beats/min, and 72–78 beats/min groups in clinical outcomes. Therefore, we dichotomized the heart rate at 80 beats/min. We found that patients with a heart rate of 80 or more beats/min had 89.4%, 115.2%, and 39.1% increased risk for ACM, CM, and MACEs, respectively, compared to patients with a heart rate less than 80 beats/min. In addition, we found that the risk increased by 2.7% in ACM, 3.0% in CM, and 1.7% in MACEs for each increase in the heart rate of 1 beat/min.

There were several strengths of our study. First, this study included a large sample size cohort, which improved the statistical power. Second, all the patients had CAD treated with PCI and experienced long-term follow-up of up to 10 years, and the follow-up duration was the longest compared to previous studies. Finally, we analyzed the data with multifaceted methods, including quintiles, bisection, and continuous variables, with heart rate, and provided a comprehensive understanding of the relationship between heart rate and clinical outcomes. However, the limitations of our study are also mentioned. First, we only collected the baseline heart rate data during the study duration. Therefore, the effect of dynamic changes in heart rate cannot be analyzed. Second, the present study is a single-center retrospective cohort design. Therefore, our results need to be further verified by a multicenter, prospective study.

## 5. Conclusion

In conclusion, the present study suggests that heart rate before PCI was an independent predictor of adverse outcomes during a 10-year follow-up. Our results emphasize the importance of targeting a low heart rate in CAD patients who have undergone PCI.

## Figures and Tables

**Table 1:** Baseline characteristics of patients.

Variables	Heart rate before PCI					*F* or *X*^2^	*P* value
	<66 beats/min	66–72 beats/min	72–78 beats/min	78–84 beats/min	≥84 beats/min		
*Total population*							
Female, *n* (%)	281 (25.0)	256 (25.3)	354 (24.5)	329 (27.2)	331 (26.2)	2.866	0.581
Smoking, *n* (%)	429 (38.2)	427 (42.3)	601 (41.7)	482 (39.8)	482 (38.2)	7.181	0.127
Drinking, *n* (%)	309 (27.5)	305 (30.2)	433 (30.0)	349 (28.8)	371 (29.4)	2.608	0.625
Diabetes, *n* (%)	217 (19.3)	196 (19.4)	362 (25.1)	308 (25.4)	368 (29.1)	45.769	**<0.001**
Hypertension, *n* (%)	440 (39.2)	404 (40.0)	594 (41.2)	520 (42.9)	598 (47.3)	20.778	**<0.001**
Age (years)	60.98 ± 10.77	59.77 ± 10.93	59.08 ± 10.74	59.43 ± 10.69	58.49 ± 10.92	8.723	**<0.001**
BMI (kg/m^2^)	25.55 ± 4.04	25.71 ± 6.89	25.73 ± 4.28	26.2 ± 10.25	26.57 ± 8.61	1.654	0.158
SBP (mmHg)	125.1 ± 19.15	125.23 ± 18.18	125.9 ± 17.14	128.52 ± 18.46	130.06 ± 20.44	16.904	**<0.001**
DBP (mmHg)	74.2 ± 11.27	74.92 ± 10.63	75.62 ± 10.32	77.05 ± 11.23	79.37 ± 12.28	40.36	**<0.001**
HR (beats/min)	60.5 ± 3.85	68.77 ± 1.55	74.31 ± 1.71	79.79 ± 1.47	91.81 ± 8.13	9115.745	**<0.001**
TG (mmol/L)	1.89 ± 1.26	1.85 ± 1.15	1.9 ± 1.27	1.91 ± 1.33	1.93 ± 1.31	0.612	0.654
TC (mmol/L)	3.95 ± 1.11	3.95 ± 1.06	3.94 ± 1.09	3.95 ± 1.11	4.01 ± 1.16	0.746	0.561
HDL-C (mmol/L)	1.0 ± 0.37	1.0 ± 0.41	1.02 ± 0.49	1.02 ± 0.47	1.05 ± 0.62	1.809	0.124
LDL-C (mmol/L)	2.43 ± 0.89	2.46 ± 0.9	2.45 ± 0.92	2.46 ± 0.9	2.5 ± 0.97	1.069	0.37
ApoA1 (mmol/L)	1.17 ± 0.31	1.16 ± 0.31	1.16 ± 0.31	1.16 ± 0.33	1.17 ± 0.32	0.202	0.938
ApoB (mmol/L)	0.85 ± 0.37	0.86 ± 0.45	0.85 ± 0.43	0.86 ± 0.42	0.85 ± 0.31	0.15	0.963
Lp(a) (mmol/L)	219.53 ± 178.71	222.2 ± 177.77	217.78 ± 170.99	216.14 ± 178.61	226.1 ± 178.94	0.58	0.677
New generation stent, *n* (%)	1060 (94.4)	951 (94.3)	1355 (94.0)	1134 (93.6)	1199 (94.9)	2.156	0.707
CTO, *n* (%)	211 (18.8)	194 (19.2)	302 (20.9)	326 (26.9)	381 (30.2)	68.650	<0.001
LM, *n* (%)	84 (7.5)	79 (7.8)	92 (6.4)	92 (7.6)	86 (6.8)	2.755	0.600
ML, *n* (%)	705 (62.8)	632 (62.6)	926 (64.2)	822 (67.9)	841 (66.6)	11.079	0.026
CCB, *n* (%)	132 (11.8)	114 (11.4)	190 (13.2)	126 (10.5)	129 (10.3)	7.592	0.108
*β*-Blockers, *n* (%)	446 (39.9)	402 (40.1)	589 (41.0)	525 (43.6)	466 (37.0)	11.274	**0.024**
ARB or ACEI, *n* (%)	232 (20.8)	235 (23.5)	320 (22.3)	284 (23.6)	296 (23.5)	3.949	0.413
Clopidogrel, *n* (%)	325 (29.1)	312 (31.3)	470 (32.8)	369 (30.7)	361 (28.8)	6.644	0.156
Aspirin, *n* (%)	769 (69.0)	725 (72.6)	963 (67.0)	801 (66.5)	793 (63.1)	24.313	**<0.001**
Statins, *n* (%)	600 (53.9)	565 (56.7)	797 (55.8)	656 (54.6)	641 (51.0)	9.205	0.056
LVEDD (mm)	50.13 ± 5.82	49.99 ± 5.58	49.77 ± 5.58	50.08 ± 5.42	49.97 ± 5.28	0.707	0.587
LVEF (%)	60.94 ± 7.18	61.09 ± 7.00	61.29 ± 6.89	61.15 ± 7.09	60.85 ± 7.06	0.687	0.601

*ACS patients*							
Female, *n* (%)	121 (24.9)	109 (24.5)	158 (27.5)	33 (29.5)	110 (25.7)	2.178	0.703
Smoking, *n* (%)	187 (38.6)	199 (44.8)	235 (40.9)	44 (39.3)	177 (41.4)	3.989	0.407
Drinking, *n* (%)	134 (27.6)	138 (31.1)	162 (28.2)	39 (34.8)	131 (30.6)	3.569	0.467
Diabetes, *n* (%)	98 (20.2)	77 (17.3)	147 (25.6)	33 (29.5)	125 (29.2)	23.683	**<0.001**
Hypertension, *n* (%)	188 (38.8)	173 (39.0)	251 (43.7)	57 (50.9)	202 (47.2)	12.464	**0.014**
Age (years)	61.01 ± 10.75	59.91 ± 10.95	59.76 ± 10.72	60.66 ± 10.74	58.51 ± 11.40	3.149	**0.014**
SBP (mmHg)	125.09 ± 19.69	126.09 ± 17.33	126.19 ± 17.03	129.14 ± 16.40	129.34 ± 21.85	3.697	**0.005**
DBP (mmHg)	73.85 ± 10.99	75.49 ± 10.56	75.72 ± 10.01	75.10 ± 8.98	80.15 ± 13.04	20.280	**<0.001**
HR (beats/min)	60.47 ± 3.98	68.81 ± 1.57	74.35 ± 1.69	78.00 ± 0.00	95.84 ± 8.69	3659.925	**<0.001**
TG (mmol/L)	1.87 ± 1.32	1.84 ± 1.15	1.93 ± 1.31	1.94 ± 1.31	2.04 ± 1.38	1.367	0.240
TC (mmol/L)	3.99 ± 1.13	3.89 ± 1.05	3.95 ± 1.06	3.91 ± 1.00	4.01 ± 1.11	0.830	0.506
HDL-C (mmol/L)	1.04 ± 0.44	1.01 ± 0.42	1.02 ± 0.43	1.01 ± 0.47	1.03 ± 0.46	0.075	0.990
LDL-C (mmol/L)	2.45 ± 0.91	2.38 ± 0.85	2.44 ± 0.89	2.51 ± 0.98	2.46 ± 0.93	0.626	0.644
ApoA1 (mmol/L)	1.17 ± 0.36	1.17 ± 0.31	1.16 ± 0.30	1.19 ± 0.52	1.18 ± 0.35	0.238	0.917
ApoB (mmol/L)	0.86 ± 0.45	0.86 ± 0.53	0.85 ± 0.44	0.85 ± 0.35	0.85 ± 0.31	0.141	0.967
Lp(a) (mmol/L)	227.91 ± 189.34	216.69 ± 156.76	213.64 ± 165.52	225.85 ± 164.24	223.11 ± 166.60	0.533	0.712
New generation stent, *n* (%)	461 (95.1)	418 (94.4)	544 (94.8)	104 (92.9)	409 (95.6)	1.602	0.808
CTO, *n* (%)	98 (20.2)	87 (19.6)	105 (18.3)	25 (22.3)	177 (41.4)	89.298	<0.001
LM, *n* (%)	44 (9.1)	40 (9.0)	46 (8.0)	13 (11.6)	39 (9.1)	1.616	0.806
ML, *n* (%)	341 (70.3)	283 (63.9)	384 (66.9)	87 (77.7)	315 (73.6)	15.182	0.004
CCB, *n* (%)	61 (12.7)	60 (13.6)	80 (14.0)	8 (7.1)	43 (10.1)	6.994	0.136
*β*-Blockers, *n* (%)	200 (41.4)	178 (40.5)	241 (42.2)	47 (42.0)	156 (36.5)	3.741	**0.442**
ARB or ACEI, *n* (%)	99 (20.5)	101 (23.0)	126 (22.1)	24 (21.4)	118 (27.6)	7.230	0.124
Clopidogrel, *n* (%)	151 (31.4)	138 (31.5)	183 (32.1)	35 (31.3)	148 (34.7)	1.534	0.821
Aspirin, *n* (%)	324 (67.5)	322 (73.5)	390 (68.3)	78 (69.6)	281 (66.0)	6.619	0.157
Statins, *n* (%)	262 (54.6)	245 (56.1)	331 (58.3)	60 (53.6)	210 (49.4)	8.109	0.088
LVEDD (mm)	50.32 ± 5.50	49.82 ± 5.48	49.84 ± 5.55	49.82 ± 4.10	49.98 ± 5.63	0.601	0.662
LVEF (%)	61.02 ± 7.09	61.25 ± 6.71	60.98 ± 6.84	62.31 ± 5.61	60.58 ± 7.25	1.345	0.251

*Stable CAD patients*							
Female, *n* (%)	160 (25.1)	147 (26.0)	196 (22.6)	296 (26.9)	221 (26.5)	5.622	0.229
Smoking, *n* (%)	242 (37.9)	228 (40.3)	366 (42.2)	438 (39.9)	305 (36.5)	6.523	0.163
Drinking, *n* (%)	175 (27.4)	167 (29.5)	271 (31.2)	310 (28.2)	240 (28.7)	3.272	0.513
Diabetes, *n* (%)	119 (18.7)	119 (21.0)	215 (24.8)	275 (25.0)	243 (29.1)	25.282	**<0.001**
Hypertension, *n* (%)	252 (39.5)	231 (40.8)	343 (39.5)	463 (42.1)	396 (47.4)	14.249	**0.007**
Age (years)	60.95 ± 10.80	59.65 ± 10.93	58.63 ± 10.75	59.31 ± 10.68	58.48 ± 10.67	5.973	**<0.001**
SBP (mmHg)	125.10 ± 18.74	124.55 ± 18.80	125.70 ± 17.22	128.46 ± 18.66	130.42 ± 19.68	13.794	**<0.001**
DBP (mmHg)	74.47 ± 11.48	74.48 ± 10.67	75.55 ± 10.52	77.24 ± 11.42	78.97 ± 11.86	22.586	**<0.001**
HR (beats/min)	60.53 ± 3.75	68.74 ± 1.54	74.28 ± 1.72	79.97 ± 1.42	89.74 ± 6.97	6480.176	**<0.001**
TG (mmol/L)	1.90 ± 1.20	1.86 ± 1.15	1.88 ± 1.24	1.91 ± 1.34	1.88 ± 1.27	0.190	0.994
TC (mmol/L)	3.92 ± 1.10	3.99 ± 1.07	3.94 ± 1.11	3.96 ± 1.12	4.01 ± 1.19	0.752	0.557
HDL-C (mmol/L)	1.00 ± 0.30	1.00 ± 0.39	1.03 ± 0.52	1.02 ± 0.46	1.06 ± 0.68	2.119	0.076
LDL-C (mmol/L)	2.41 ± 0.87	2.52 ± 0.93	2.45 ± 0.93	2.45 ± 0.89	2.52 ± 0.98	1.915	0.105
ApoA1 (mmol/L)	1.17 ± 0.27	1.15 ± 0.31	1.17 ± 0.31	1.16 ± 0.31	1.16 ± 0.31	0.361	0.836
ApoB (mmol/L)	0.83 ± 0.29	0.86 ± 0.38	0.85 ± 0.42	0.85 ± 0.42	0.85 ± 0.31	0.497	0.738
Lp(a) (mmol/L)	213.26 ± 170.24	226.54 ± 192.78	220.48 ± 174.53	215.14 ± 180.06	227.58 ± 184.83	0.928	0.446
New generation stent, *n* (%)	599 (93.9)	533 (94.2)	811 (93.4)	1030 (93.7)	790 (94.6)	1.198	0.878
CTO, *n* (%)	113 (17.7)	107 (18.9)	197 (22.7)	301 (27.4)	204 (24.4)	28.384	<0.001
LM, *n* (%)	40 (6.3)	39 (6.9)	46 (5.3)	79 (7.2)	47 (5.6)	3.926	0.416
ML, *n* (%)	364 (57.1)	349 (61.7)	542 (62.4)	735 (66.9)	526 (63.0)	17.222	0.002
CCB, *n* (%)	71 (11.2)	54 (9.6)	110 (12.7)	118 (10.8)	86 (10.3)	4.203	0.379
*β*-Blockers, *n* (%)	246 (38.7)	224 (39.8)	348 (40.2)	478 (43.7)	310 (37.3)	9.162	**0.057**
ARB or ACEI, *n* (%)	133 (20.9)	134 (23.8)	194 (22.4)	260 (23.8)	178 (21.4)	3.126	0.537
Clopidogrel, *n* (%)	174 (27.4)	174 (31.1)	287 (33.3)	334 (30.6)	213 (25.7)	14.202	0.007
Aspirin, *n* (%)	445 (70.1)	403 (71.8)	573 (66.2)	723 (66.2)	512 (61.7)	19.536	**0.001**
Statins, *n* (%)	338 (53.3)	320 (57.2)	466 (54.2)	596 (54.7)	431 (51.9)	4.231	**0.376**
LVEDD (mm)	49.98 ± 6.06	50.12 ± 5.67	49.73 ± 5.59	50.10 ± 5.53	49.96 ± 5.09	0.590	0.670
LVEF (%)	60.88 ± 7.26	60.97 ± 7.23	61.48 ± 6.92	61.03 ± 7.21	60.99 ± 6.96	0.801	0.524

ACEI, angiotensin-converting enzyme inhibitor; ARB, angiotensin receptor blocker; BMI, body mass index; SBP, systolic blood pressure; DBP, diastolic blood pressure; HR, heart rate; TG, triglyceride; TC, total cholesterol; LDL-C, low-density lipoprotein cholesterol; HDL-C, high-density lipoprotein cholesterol; ApoA1, apolipoprotein A1; ApoB, apolipoprotein B; Lp(a), lipoprotein a; CTO, chronic total occlusion lesions; LM, left main lesions; ML, multivessel lesions; LVEDD, left ventricular end diastolic diameter; LVEF, left ventricular ejection fraction.

**Table 2:** Clinical outcomes and heart rates according to quintiles.

Variables	Heart rate before PCI					*X* ^2^	*P* value
	<66 beats/min	66–72 beats/min	72–78 beats/min	78–84 beats/min	≥84 beats/min		
Total patients							
ACM	43 (3.8)	38 (3.8)	62 (4.3)	77 (6.4)	89 (7.0)	23.206	**<0.001**
CM	37 (3.3)	26 (2.6)	48 (3.3)	60 (5.0)	80 (36)	27.936	**<0.001**
MACEs	137 (12.2)	112 (11.1)	172 (11.9)	181 (14.9)	183 (14.5)	11.911	**0.018**
Stroke	13 (1.2)	13 (1.3)	22 (1.5)	18 (1.5)	16 (1.3)	0.906	0.924
Readmission	155 (13.8)	130 (12.9)	198 (13.7)	178 (14.7)	158 (12.5)	3.03	0.553
Bleeding events	39 (3.5)	25 (2.5)	43 (3.0)	35 (2.9)	33 (2.6)	2.365	0.669
ACS patients							
ACM	23 (4.7)	11 (2.5)	17 (3.0)	5 (4.5)	32 (7.5)	16.788	**0.002**
CM	21 (4.3)	8 (1.8)	13 (2.3)	3 (2.7)	29 (6.8)	20.418	**<0.001**
MACEs	70 (14.4)	43 (9.7)	67 (11.7)	17 (15.2)	70 (16.4)	10.763	**0.029**
Stroke	2 (0.4)	5 (1.1)	9 (1.6)	5 (4.5)	7 (1.6)	11.808	**0.019**
Readmission	70 (14.4)	59 (13.3)	87 (15.2)	18 (16.1)	58 (13.6)	1.205	0.887
Bleeding events	18 (3.7)	13 (2.9)	12 (2.1)	1 (0.9)	11 (2.6)	4.219	0.377
Stable CAD patients							
ACM	20 (3.1)	27 (4.8)	45 (5.2)	72 (6.6)	57 (6.8)	12.738	**0.013**
CM	16 (2.5)	18 (3.2)	35 (4.0)	57 (5.2)	51 (6.1)	15.054	**0.005**
MACEs	67 (10.5)	69 (12.2)	105 (12.1)	164 (14.9)	113 (13.5)	8.297	0.081
Stroke	11 (1.7)	8 (1.4)	13 (1.5)	13 (1.2)	9 (1.1)	1.527	0.822
Readmission	85 (13.3)	71 (12.5)	111 (12.8)	160 (14.6)	100 (12.0)	3.214	0.523
Bleeding events	21 (3.3)	12 (2.1)	31 (3.6)	34 (3.1)	22 (2.6)	3.086	0.543

**Table 3:** Multivariable Cox regression analysis of ACM.

Variables	*B*	SE	Wald	*P* values	HR (95% CI)
Age	0.021	0.006	12.362	<0.001	1.021 (1.009–1.032)
SBP	−0.004	0.004	1.218	0.270	0.996 (0.988–1.003)
DBP	−0.001	0.006	0.045	0.832	0.999 (0.986–1.011)
Diabetes	0.049	0.147	0.112	0.737	1.051 (0.787–1.402)
Hypertension	0.162	0.132	1.490	0.222	1.175 (0.907–1.524)
ML	0.481	0.136	12.487	<0.001	1.618 (1.239–2.112)
CTO	0.288	0.145	3.962	0.047	1.333 (1.004–1.770)
Aspirin	−1.955	0.203	92.801	<0.001	0.142 (0.095–0.211)
Statins	−1.008	0.232	18.879	<0.001	0.365 (0.232–0.575)
*β*-Blockers	−0.905	0.237	14.624	<0.001	0.405 (0.254–0.643)
LVEDD	−0.015	0.012	1.579	0.209	0.985 (0.962–1.008)
LVEF	−0.006	0.009	0.354	0.552	0.994 (0.976–1.013)
Heart rate (<66 beats/min as reference)			21.329	<0.001	
66–72 beats/min	0.124	0.236	0.275	0.600	1.132 (0.713–1.796)
72–78 beats/min	0.112	0.214	0.274	0.601	1.119 (0.735–1.703)
78–84 beats/min	0.583	0.201	8.388	0.004	1.791 (1.207–2.657)
≥84 beats/min	0.686	0.199	11.849	0.001	1.987 (1.344–2.937)

**Table 4:** Multivariable Cox regression analysis of CM.

Variables	*B*	SE	Wald	*P* values	HR (95% CI)
Age	0.014	0.007	4.577	0.032	1.014 (1.001–1.027)
SBP	−0.003	0.004	0.354	0.552	0.997 (0.989–1.006)
DBP	−0.004	0.007	0.256	0.613	0.996 (0.982–1.011)
Diabetes	0.119	0.162	0.536	0.464	1.126 (0.820–1.547)
Hypertension	0.126	0.148	0.720	0.396	1.134 (0.848–1.516)
ML	0.565	0.151	14.003	<0.001	1.759 (1.309–2.365)
CTO	0.269	0.162	2.756	0.097	1.309 (0.953–1.798)
Aspirin	−1.914	0.220	75.373	<0.001	0.147 (0.096–0.227)
Statins	−0.891	0.247	13.003	<0.001	0.410 (0.253–0.666)
*β*-Blockers	−0.799	0.252	10.039	0.002	0.450 (0.274–0.737)
LVEDD	−0.020	0.013	2.256	0.133	0.980 (0.955–1.006)
LVEF	−0.015	0.010	2.238	0.135	0.985 (0.965–1.005)
Heart rate (<66 beats/min as reference)			25.145	<0.001	
66–72 beats/min	−0.186	0.275	0.459	0.498	0.830 (0.485–1.423)
72–78 beats/min	−0.090	0.239	0.142	0.707	0.914 (0.572–1.461)
78–84 beats/min	0.451	0.220	4.186	0.041	1.569 (1.019–2.416)
≥84 beats/min	0.687	0.213	10.423	0.001	1.988 (1.310–3.016)

**Table 5:** Multivariable Cox regression analysis of MACEs.

Variables	*B*	SE	Wald	*P* value	HR (95% CI)
Age	−0.001	0.004	0.153	0.696	0.999 (0.991–1.006)
SBP	−0.002	0.002	0.834	0.361	0.998 (0.993–1.003)
DBP	0.003	0.004	0.373	0.541	1.003 (0.994–1.011)
Diabetes	0.162	0.090	3.246	0.072	1.176 (0.986–1.402)
Hypertension	0.291	0.082	12.565	0.000	1.338 (1.139–1.572)
ML	0.249	0.089	7.860	0.005	1.283 (1.078–1.527)
CTO	0.311	0.089	12.232	0.000	1.364 (1.146–1.624)
Aspirin	−0.551	0.090	37.720	0.000	0.577 (0.484–0.687)
Statins	−0.008	0.095	0.007	0.934	0.992 (0.824–1.195)
*β*-Blockers	−0.086	0.092	0.869	0.351	0.918 (0.766–1.099)
LVEDD	−0.013	0.008	2.674	0.102	0.987 (0.972–1.003)
LVEF	−0.006	0.006	0.878	0.349	0.994 (0.982–1.006)
Heart rate (<66 beats/min as reference)			14.798	0.005	
66–72 beats/min	−0.015	0.135	0.012	0.913	0.985 (0.756–1.284)
72–78 beats/min	−0.057	0.123	0.212	0.646	0.945 (0.742–1.203)
78–84 beats/min	0.227	0.121	3.532	0.060	1.255 (0.990–1.590)
≥84 beats/min	0.309	0.122	6.365	0.012	1.361 (1.071–1.730)

**Table 6:** Clinical outcomes and heart rate as continuous variable and bisection.

Outcomes	HR (95% CI)	*P* values	Adjusted HR (95% CI)	*P* values
Total population				
ACM				
Heart rate ≥80 vs. <80 beats/min	1.894 (1.513–2.370)	<0.001	1.905 (1.507–2.408)	<0.001
Heart rate higher by 1 beat/min	1.027 (1.018–1.037)	<0.001	1.027 (1.018–1.037)	<0.001
CM				
Heart rate ≥80 vs. <80 beats/min	2.152 (1.680–2.757)	<0.001	2.170 (1.672–2.815)	<0.001
Heart rate higher by 1 beat/min	1.030 (1.020–1.040)	<0.001	1.030 (1.020–1.041)	<0.001
MACEs				
Heart rate ≥80 vs. <80 beats/min	1.391 (1.203–1.607)	<0.001	1.373 (1.181–1.596)	<0.001
Heart rate higher by 1 beat/min	1.017 (1.011–1.023)	<0.001	1.015 (1.009–1.022)	<0.001
ACS patients				
ACM				
Heart rate ≥80 vs. <80 beats/min	2.668 (1.726–4.121)	<0.001	2.689 (1.661–4.352)	<0.001
Heart rate higher by 1 beat/min	1.025 (1.010–1.039)	<0.001	1.026 (1.010–1.042)	0.001
CM				
Heart rate ≥80 vs. <80 beats/min	2.993 (1.876–4.778)	<0.001	3.195 (1.894–5.390)	<0.001
Heart rate higher by 1 beat/min	1.026 (1.010–1.042)	0.001	1.029 (1.011–1.046)	0.001
MACEs				
Heart rate ≥80 vs. <80 beats/min	1.686 (1.283–2.215)	<0.001	1.704 (1.283–2.263)	<0.001
Heart rate higher by 1 beat/min	1.013 (1.004–1.022)	0.004	1.012 (1.003–1.022)	0.013
Stable CAD patients				
ACM				
Heart rate ≥80 vs. <80 beats/min	1.570 (1.206–2.044)	0.001	1.573 (1.198–2.066)	0.001
Heart rate higher by 1 beat/min	1.030 (1.018–1.043)	<0.001	1.030 (1.017–1.42)	<0.001
CM				
Heart rate ≥80 vs. <80 beats/min	1.809 (1.347–2.431)	<0.001	1.819 (1.339–2.471)	<0.001
Heart rate higher by 1 beat/min	1.035 (1.021–1.049)	<0.001	1.035 (1.021–1.049)	<0.001
MACEs				
Heart rate ≥80 vs. <80 beats/min	1.273 (1.070–1.515)	0.007	1.248 (1.051–1.483)	0.011
Heart rate higher by 1 beat/min	1.019 (1.011–1.028)	<0.001	1.019 (1.010–1.028)	<0.001

## Data Availability

The data used to support the findings of this study are available from the corresponding author upon request.
